# Moderated mediation model of basic needs supports, intrinsic motivation, and PERMA well-being among college students in physical education: the moderating roles of exercise causality orientations

**DOI:** 10.3389/fpsyg.2026.1774745

**Published:** 2026-03-05

**Authors:** Liang Han, Man-man Li, Chong-yao Xiao

**Affiliations:** 1 Department of Social Sports, Henan Sport University, Zhengzhou, China; 2 School of Physical Education and Sport, Henan University, Kaifeng, China; 3 School of Physical Education, Zhengzhou Normal University, Zhengzhou, China; 4 School of Education and Health, Guilin Institute of Information Technology, Guilin, China

**Keywords:** causality orientation, intrinsic motivation, moderated mediation model, needs support, PERMA well-being, physical education

## Abstract

**Introduction:**

Based on self-determination theory, this study examined the mediating role of intrinsic motivation in the relationship between needs support and PERMA well-being (positive emotion, engagement, relationships, meaning, and accomplishment) among college students in physical education. Furthermore, this study investigated the moderating roles of exercise causality orientations in the mediation model.

**Methods:**

Undergraduate students completed questionnaires with demographic information, the Needs Support Scale, Intrinsic Motivation Inventory, and Exercise Causality Orientations Scale. Data analyses were performed on data from 626 valid questionnaires to determine the relationship between the variables.

**Results:**

The results demonstrated that the three causality orientations moderated the relationship between needs support and intrinsic motivation in different ways. Specifically, autonomy orientation strengthened the positive predictive effect of needs support on intrinsic motivation, control orientation had no moderating effect, and impersonal orientation weakened the positive association between needs support and intrinsic motivation-showing a negative predictive trend. The findings indicated that needs support may not always positively predict intrinsic motivation, especially unilaterally providing needs support is not associated with enhanced intrinsic motivation among individuals with high impersonal orientation.

**Discussion:**

Thus, students with an impersonal orientation should be guided toward an autonomy orientation. This study provides insights into the interactive influence of environmental factors and personal characteristics on motivation and PERMA well-being in physical education.

## 1 Introduction

Chinese university students exhibit a higher prevalence of psychological distress (e.g., depression, anxiety) than the global average (Huang et al., 2023). Given the robust link between mental health and holistic well-being (Alsamiri et al., 2024; Saijonkari et al., 2023), this study targets well-being as a pathway to ameliorate college students’ psychological outcomes. Traditional unidimensional conceptualizations of well-being (either hedonic pleasure or eudaimonic purpose; Ryan and Deci, 2001) restrict intervention efficacy, whereas a dual-focus on both hedonia and eudaimonia enables multifaceted well-being experiences (Braaten et al., 2019).

Yet few studies have applied the PERMA model (positive emotion, engagement, relationships, meaning, accomplishment)—a multidimensional framework integrating hedonic and eudaimonic well-being (Seligman, 2012)—to Chinese college students, especially in mandatory physical education (PE), where interactive and skill-building contexts inherently align with PERMA dimensions (Kovich et al., 2023; Yang and Mohd, 2021). PERMA’s congruence with self-determination theory (SDT) core tenets (need satisfaction and intrinsic motivation drive holistic flourishing; Ryan and Deci, 2020) highlights a critical gap: current PE research lacks frameworks linking instructional need support, motivation, and multidimensional well-being (Standage et al., 2005).

Despite progress in SDT-based PE research, most studies only examine main effects of teacher need support, ignoring how students’ dispositional motivational styles shape their responses to such support (McAnally and Hagger, 2024). Causality Orientations Theory (COT; Deci and Ryan, 1985) posits three stable motivational orientations (autonomy, control, impersonal) that guide environment interactions and may moderate environment-support–psychological outcome links (Ryan and Deci, 2017), yet empirical evidence for these interactive effects in PE remains scarce.

**Figure 1:**
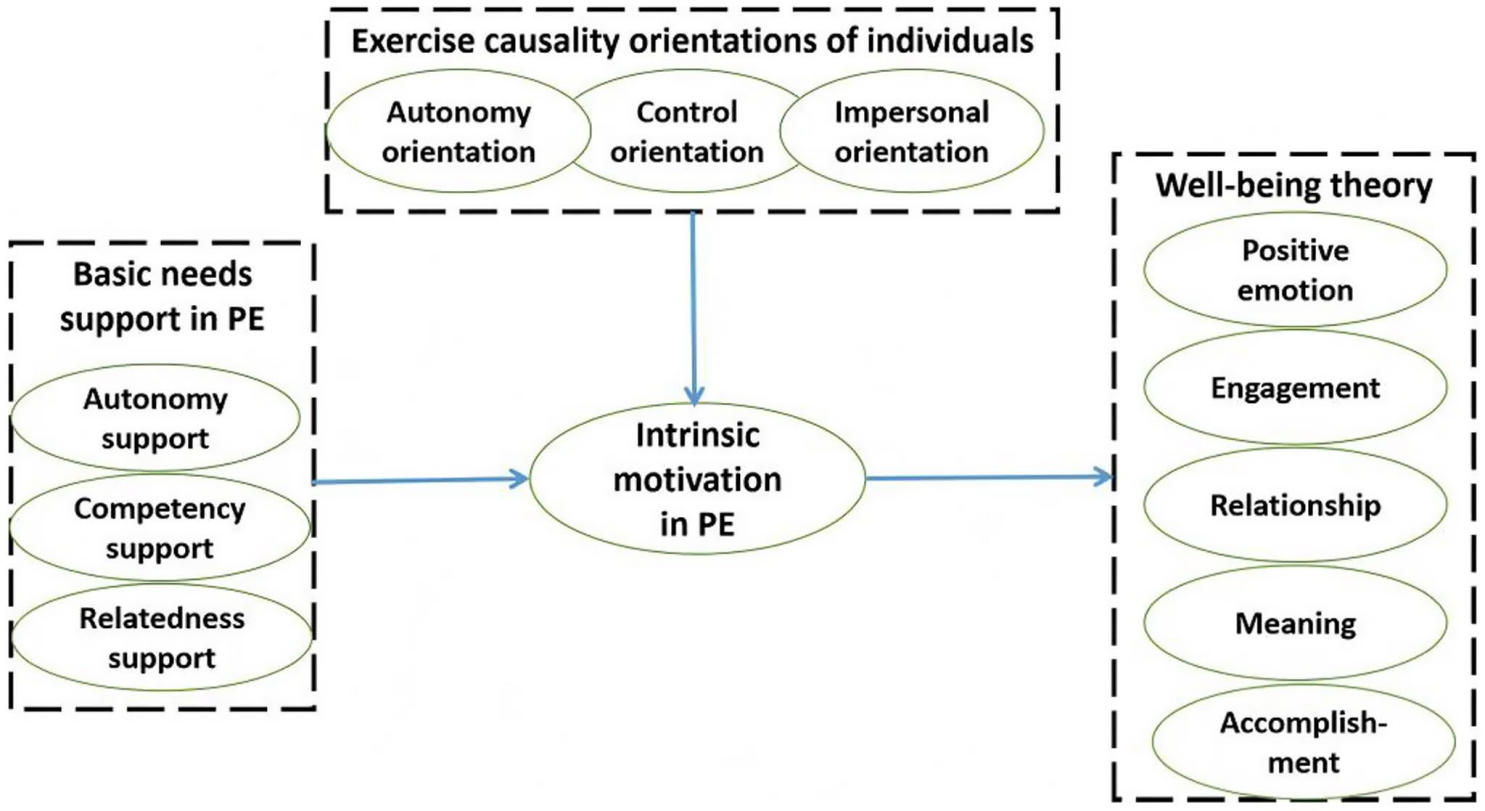
Theoretical model of this study.

This study tests a moderated mediation model (Figure 1) to clarify whether the efficacy of need support in promoting PERMA well-being via intrinsic motivation is universal or contingent on students’ exercise causality orientations. By integrating SDT and COT in mandatory PE contexts, we explore person-environment interactions to identify for whom and how this motivational pathway operates optimally, aiming to inform tailored well-being interventions.

**Figure 2:**
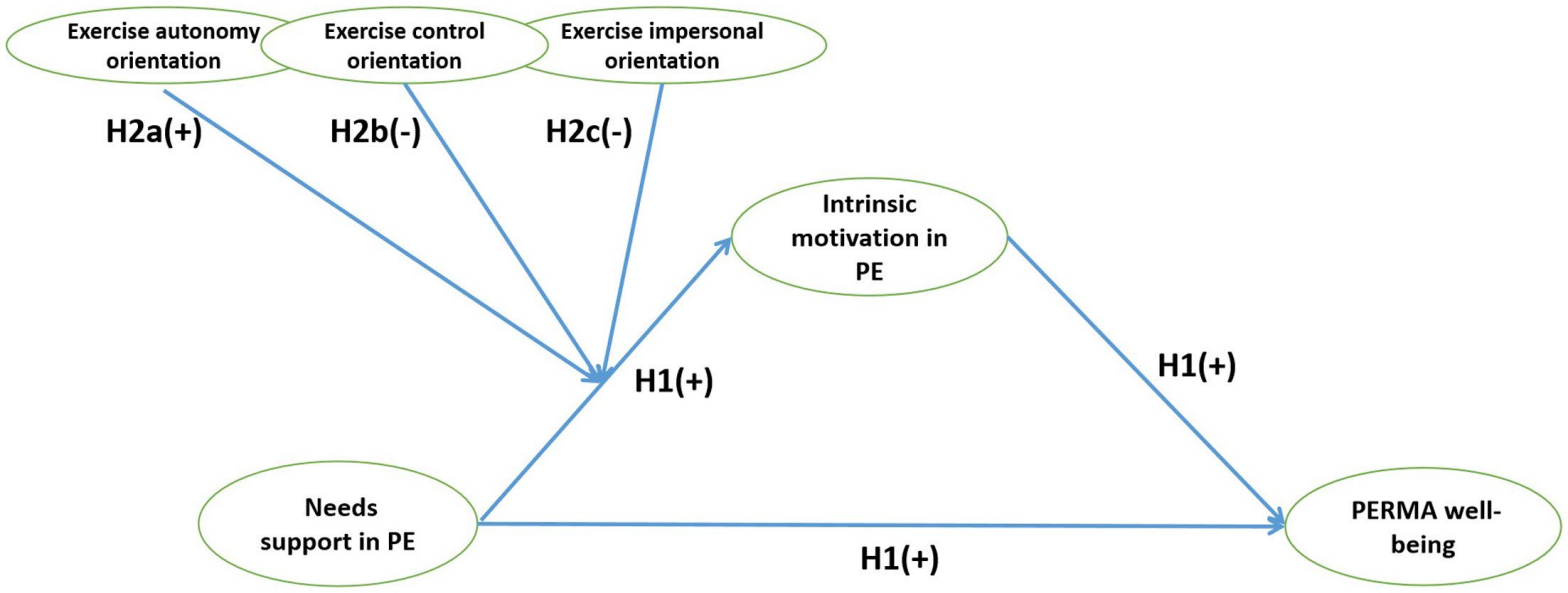
The overall hypothetical model.

In PE, autonomy-supportive teaching consistently enhances intrinsic motivation and adaptive outcomes (Abula et al., 2020; Wang et al., 2024). Intrinsic motivation—characterized by inherent enjoyment—predicts engagement and sustained physical activity (Manninen et al., 2022). The PERMA model provides a comprehensive framework for assessing well-being with growing application in higher education (Bonneville-Roussy et al., 2020; Wang et al., 2024). However, the mediating role of intrinsic motivation between PE teacher need support and PERMA well-being remains underestablished. We thus propose:

*H1*: PE teachers’ needs support positively predicts students’ PERMA well-being through the mediating role of their intrinsic motivation (Figure 2).

The mediation pathway proposed in H1, however, may not universally apply to all students. This assumption of uniformity overlooks individual differences in motivational dispositions, as outlined by COT (Deci and Ryan, 1985). COT posits that individuals possess relatively stable motivational styles—autonomy, control, and impersonal orientations—that shape how they perceive and interact with environmental supports (Ryan and Deci, 2017). Crucially, these orientations may moderate how students respond to teachers’ need-supportive behaviors, yet empirical tests of these interactive effects in PE contexts remain scarce (Manninen and Campbell, 2021; McAnally and Hagger, 2024).

To address this gap, we tested a moderated mediation model to examine whether the strength of the entire mediation process (from needs support to intrinsic motivation to PERMA well-being) is contingent upon a student’s causality orientation. We derive specific hypotheses based on the characteristics of each orientation:

*Autonomy orientation*: Students with a strong autonomy orientation act out of interest and value congruence. They are likely to perceive needs support as an opportunity for self-endorsed action, thereby actively engaging with it and amplifying its positive effect on intrinsic motivation (Wang et al., 2024). Thus, we hypothesize:

*H2a*: Autonomy orientation positively moderates the relationship between needs support and intrinsic motivation (Figure 2).

*Control orientation*: Students with a high control orientation are primarily driven by external contingencies such as rewards and evaluations. Consequently, while they may respond to needs support, its effect on fostering the more self-determined and spontaneous intrinsic motivation is likely to be attenuated (Slemp et al., 2020). Therefore, we propose:

*H2b*: Control orientation negatively moderates the relationship between needs support and intrinsic motivation (Figure 2).

*Impersonal orientation*: Characterized by perceived incompetence and learned helplessness, impersonal orientation can lead students to misinterpret teachers’ supportive behaviors as external pressure, triggering need frustration and undermining intrinsic motivation (Behzadnia, 2020). Prior research further shows this orientation may reverse the benefits of environmental support, generating counterproductive outcomes (Souesme et al., 2020). We therefore hypothesize:

*H2c*: Impersonal orientation negatively moderates the relationship between needs support and intrinsic motivation (Figure 2).

In summary, this study proposes a moderated mediation model integrating SDT, COT, and the PERMA well-being framework. Grounded in SDT, the model examines how the environmental factor of teachers’ need support enhances the multifaceted outcome of PERMA well-being through the psychological mechanism of intrinsic motivation. We integrate COT to test whether this entire motivational sequence is moderated by students’ dispositional causality orientations. This integration allows us to move beyond asking if need support works, to investigate for whom and under what conditions it is most effective in promoting multifaceted well-being, thereby testing a crucial boundary condition of SDT in a mandatory educational setting.

## 2 Materials and methods

### 2.1 Participants and procedure

At Chinese universities, PE is a compulsory course during the first 2 years of undergraduate study. For this study, questionnaires were collected from second- and third-year undergraduate students. First-year students were excluded because they had relatively limited experience with PE at the time of the survey, and fourth-year students were excluded because they typically select very few PE courses as they prepare for their graduation.

This study employed a two-stage stratified random sampling method. First, to ensure regional and institutional diversity, we selected three provinces (Henan, Guangdong, Jiangsu) representing central, southern, and eastern China. Within each province, universities were stratified by type (public/private) and academic focus (comprehensive, science and technology, normal). From these strata, a total of 10 universities were randomly selected (three from Henan, three from Guangdong, and four from Jiangsu). Second, within each university, majors were stratified into three categories: arts and humanities, sciences and engineering, and social sciences and business. From each category, two to three classes were randomly selected, and all second- and third-year undergraduates in these classes were invited to participate.

To recruit participants, we initially contacted the administrators and PE department heads at each university. They helped identify eligible classes and informed the students about the study. Questionnaires were distributed via an online questionnaire platform (Qualtrics) between March 1 and May 1, 2024, during the spring semester. The link was sent to students via their university email addresses or online learning management systems, and a reminder email was sent 1 week after the initial invitation to boost the response rate.

The online questionnaire consisted of several sections. The first part collected demographic details such as age (ranging from 18 to 22), sex (306 male participants, accounting for 48.9%, and 320 female participants, accounting for 51.1%), year of study (284 second-year students, 45.4%, and 342 third-year students, 54.6%), major (covering diverse academic disciplines), and region (representing different geographical areas within the three provinces). The subsequent sections evaluated students’ experiences with PE teachers’ needs support (using the Needs Support Scale), exercise causality orientations (assessed by the Exercise Causality Orientations Scale), intrinsic motivation (measured by the Intrinsic Motivation Inventory), and PERMA well-being (evaluated with the PERMA-Profiler).

Before commencing the survey, participants were presented with a comprehensive introduction that detailed the study’s purpose, the estimated time to complete the questionnaire (approximately 20 min), and the assurance of confidentiality and anonymity of their responses. They were informed that their participation was voluntary and that they could skip any questions they preferred not to answer. This study was reviewed and approved by the Ethics Committee of Henan Sport University. All procedures were conducted in accordance with the ethical standards of the institutional and/or national research committee and with the 1964 Helsinki Declaration and its later amendments or comparable ethical standards. Participants indicated their consent by clicking an “I Agree” button before proceeding with the questionnaire. The questionnaire was designed to prevent submission of incomplete responses, and participants could review and modify their answers before final submission.

We initially received 1,029 responses. The data cleaning procedure was conducted as follows, with thresholds set based on methodological conventions and pilot testing: First, 187 responses with a completion time of less than 5 min were excluded, a criterion determined by a pilot study to flag insincere or rushed responses. Second, 65 cases exhibiting substantial missing data (>10% of items across all scales) were removed, applying a common benchmark to preserve dataset integrity. For the remaining dataset, the minimal random missing values (affecting less than 0.5% of all data points) were imputed using the Expectation–Maximization (EM) algorithm, which is appropriate for handling data missing at random. Finally, 151 multivariate outliers were identified and removed using Mahalanobis distance with a stringent criterion of *p* < 0.001. After these steps, 626 valid questionnaires remained, yielding a response rate of 60.83%.

### 2.2 Demographic characteristics

The demographic profile of the final valid sample (*N* = 626) is summarized in Table 1. The sample comprised a relatively balanced distribution of male (48.9%) and female (51.1%) students, primarily in their second (45.4%) and third (54.6%) academic years, with a mean age of 20.1 years (SD = 1.3). Participants were drawn from universities in three provinces and represented diverse academic majors.

**Table 1:** Demographic characteristics of participants (*N* = 626).

Characteristic	Category	Frequency (*n*)	Percentage (%)
Sex	Male	306	48.9%
	Female	320	51.1%
Academic year	Second-year	284	45.4%
	Third-year	342	54.6%
Age (years)	Mean (SD)	20.1 (1.3)	
	Range	18–22	
Region	Henan	245	39.1%
	Guangdong	195	31.2%
	Jiangsu	186	29.7%
Major field	Arts and humanities	158	25.2%
	Sciences and engineering	245	39.1%
	Social sciences and business	223	35.6%

### 2.3 Measures

#### 2.3.1 Needs support

We used the Needs Support Scale developed by Standage et al. (2005). The scale comprises three dimensions: autonomy (e.g., “The PE teacher encourages us to ask questions during PE.”), competence, and relatedness support. Responses are rated on a seven-point Likert scale (1 = *strongly disagree;* 7 = *strongly agree*). Standage et al. (2005) demonstrated the reliability and validity of this scale and its subscales. Moreover, this scale has been used in China and has shown good internal reliability (Cronbach’s *α* > 0.87) (Yin, 2019). In the current sample, Cronbach’s *α* was 0.94 (Table 2).

**Table 2:** Means, standard deviations (SDs), and correlation coefficients between variables.

Variable	Cronbach’s *α*	M	SD	1	2	3	4	5	6
1 NsSu	0.942	5.715	0.981	1					
2 IM	0.858	4.401	0.374	0.519***	1				
3 PERMA	0.963	7.027	1.558	0.408***	0.487***	1			
4 AO	0.734	4.853	0.79	0.338***	0.431***	0.312***	1		
5 CO	0.909	3.558	1.345	−0.685***	−0.807***	−0.461***	−0.439***	1	
6 IO	0.872	3.589	1.239	−0.752***	−0.808***	−0.483***	−0.405***	0.852***	1

NsSu, needs support; IM, intrinsic motivation; PERMA, PERMA well-being; AO, autonomy orientation; CO, control orientation; IO, impersonal orientation.

***p < 0.001.

#### 2.3.2 Intrinsic motivation

This study used the Intrinsic Motivation Inventory (IMI) for PE compiled and validated by Tsigilis and Theodosiou (2003) based on the original scale developed by Ryan and Deci (2000). The IMI comprises 18 items in four dimensions: enjoyment/interest (e.g., “What we do in PE is very interesting”), stress/strain, effort/importance, and perceived competence. Responses are rated on a seven-point Likert scale (1 = *strongly disagree*; 7 = *strongly agree*). Previous research has demonstrated the scale’s internal consistency (Cronbach’s *α* = 0.85). Furthermore, the scale has been frequently used in sports programs in China and has shown good reliability and validity (Xiong and Ma, 2013). In this study, its internal consistency was 0.86 (Table 2).

#### 2.3.3 PERMA well-being

PERMA well-being was measured using the PERMA-Profiler developed by Butler and Kern (2016). In addition to the four dimensions of overall well-being, loneliness, physical health, and negative emotions, the scale contains the five dimensions of PERMA: positive emotions, engagement (e.g., “How often do you become absorbed in what you are doing?”), relationships, meaning, and accomplishment. This study used three items from each of these five dimensions, for a total of 15 items. Responses to these items were measured using an 11-point Likert scale, where 0 represents “*never/not at all”* and 10 represents “*always/completely*.” The Chinese version of the PERMA-Profiler demonstrated satisfactory reliability and validity (Cronbach’s α = 0.95) (Lai et al., 2018). In the present sample, Cronbach’s *α* was 0.96 (Table 2).

#### 2.3.4 Causality orientations

Deci and Ryan (1985) argued that to accurately predict behavior in certain domains, the General Causality Orientations Scale must be assessed in specific contexts, as different outcomes influenced by environmental factors require a precise evaluation of orientations. The Exercise Causality Orientations Scale, developed by Rose et al. (2001), was designed for the exercise environment based on the General Causality Orientations Scale. The Exercise Causality Orientations Scale contains seven scenarios in an exercise environment (e.g., “You are starting a new exercise program. You are likely to?”), each comprising three questions with different response styles corresponding to autonomy (e.g., “Decide for yourself which type of exercise you would like to complete”), control (e.g., “Attend a structured exercise class where an exercise leader is telling you what to do”), and impersonal (e.g., “Tag along with your friends and do what they do”) orientations. Responses are rated on a seven-point Likert scale (1 = *very unlikely*; 7 = *very likely*). Orientation scores are determined by calculating the total mean of the orientations. Higher scores indicate the individual’s dominant orientation. This scale has been applied in China and has shown acceptable internal consistency, with Cronbach’s *α* coefficients for the three subscales and total scale of 0.772, 0.650, 0.636, and 0.798, respectively (Ding and Mao, 2014). In the present study, the internal consistency estimates for the autonomy, control, and impersonal orientation subscales were 0.73, 0.91, and 0.87, respectively (Table 2).

### 2.4 Data analysis

This study used non-experimental quantitative methods to explore theoretical conceptual models and explain existing, directly observable phenomena. The data were processed and tested using SPSS 26.0 and Amos 26.0. Pearson correlation analyses were conducted on needs support, PERMA well-being, intrinsic motivation, and causality orientations. PROCESS macro in SPSS, developed by Hayes (2017), was used, with needs support as the independent variable, PERMA well-being as the dependent variable, and intrinsic motivation as the mediating variable. The nonparametric percentile bootstrap method with bias correction was used to test the hypotheses. If the 95% confidence interval (CI) of the mean path coefficient does not contain zero, the mediating effect is significant; if it contains 0, it is not significant (Preacher et al., 2007).

## 3 Results

### 3.1 Preliminary analyses

The confirmatory factor analysis model fit results for each variable met the criteria, indicating good structural validity (Table 3). As all the measurement data were self-reported, procedural controls (e.g., item reversal, anonymity assurance) were implemented. To comprehensively assess potential common method bias (CMB), two complementary tests were conducted. First, Harman’s single-factor test extracted eight factors with eigenvalues greater than one, with the largest factor explaining 31.2% of the variance, below the 40% threshold. Second, a more stringent confirmatory factor analysis (CFA) was performed. A model forcing all items to load on a single common factor showed poor fit (*χ*^2^/df = 5.86, CFI = 0.67, TLI = 0.65, RMSEA = 0.089). This fit was significantly worse than that of the hypothesized five-factor measurement model (*χ*^2^/df = 2.91, CFI = 0.94, TLI = 0.93, RMSEA = 0.062; Δ*χ*^2^ (15) = 624.71, *p* < 0.001). Together, these results indicate that significant common method bias is unlikely in this study. Before testing the main hypotheses, we examined the data to assess the assumptions of regression analysis. The variance inflation factor (VIF) for all predictors ranged from 1.5 to 2.8, well below the threshold of 5, indicating that multicollinearity was not a concern. Visual inspection of residual plots also suggested that the assumptions of normality and homoscedasticity were reasonably met.

**Table 3:** Confirmatory factor analysis results.

Variables	*χ* ^2^	df	*p*	*χ*^2^/df	CFI	GFI	IFI	TLI	RMSEA
Fit standard	——	——	——	0–10	>0.9	>0.9	>0.9	>0.9	0–0.1
COs	476.359	186	0.000	2.561	0.952	0.926	0.952	0.946	0.05
IM	711.825	102	0.000	6.979	0.936	0.901	0.936	0.904	0.098
NsSu	510.664	82	0.000	6.228	0.967	0.905	0.967	0.958	0.091
PERMA	428.893	77	0.000	5.57	0.963	0.913	0.963	0.949	0.086

COs, causality orientations; IM, intrinsic motivation; NsSu, needs support; PERMA, PERMA well-being.

### 3.2 Descriptive statistics and correlations

Autonomy orientation was positively correlated with needs support, intrinsic motivation, and PERMA well-being. Control orientation was negatively correlated with needs support, intrinsic motivation, and PERMA well-being. Impersonal orientation was negatively correlated with needs support, intrinsic motivation, and PERMA well-being (Table 2). The correlation patterns were consistent with our theoretical model, providing preliminary support for the hypothesized relationships.

### 3.3 Mediating effect

**Figure 3:**
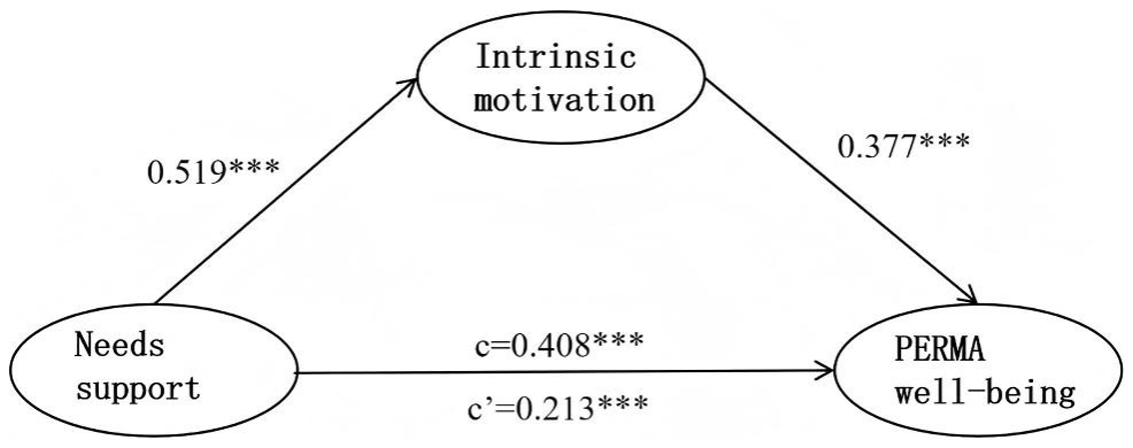
Diagram of the mediating effect test model.

Model 4 was selected for mediating effect testing based on PROCESS templates: Unmediated Model 1 (Y = cX + e1) for the independent and dependent variables, Model 2 (M = aX + e2) for the independent and mediating variables, and Model 3 (Y = dX + bM + e3) with mediating variables. The results are presented in Table 4 and Figure 3.

**Table 4:** Mediating effect of intrinsic motivation in the relationship between needs support and PERMA well-being.

Model	Regression equation		Model fit		Coefficient	
	Outcome variable	Predictor	*R* ^2^	*F*	*β*	*t*
1	PERMA	NsSu	0.167	124.730***	0.408	11.168***
2	IM	NsSu	0.269	229.610***	0.519	15.153***
3	PERMA	NsSu	0.271	115.551***	0.213	5.311***
		IM			0.377	9.424***

NsSu, needs support; IM, intrinsic motivation; PERMA, PERMA well-being.

***p < 0.001.

The independent variable significantly and positively predicted the dependent variable. After adding the mediating variable, the independent variable continued to significantly and positively predict the dependent variable. Furthermore, the independent variable significantly predicted the mediating variable, and the mediating variable significantly predicted the dependent variable.

The mediating variable partially mediated the relationship between the independent and dependent variables (Table 5). Thus, needs support was associated with PERMA well-being both directly and indirectly through intrinsic motivation, supporting H1.

**Table 5:** Decomposition of total, mediating, and direct effects.

Decomposition	Effect	Boot SE	95% CI		Ratio
			Lower limit	Upper limit	
Total	0.408	0.058	0.534	0.762	
Direct	0.213	0.064	0.213	0.462	52.06%
Indirect	0.196	0.025	0.147	0.246	47.92%

Supporting H1, the bootstrap results confirmed a significant partial mediation model, as the indirect effect of needs support on PERMA well-being through intrinsic motivation was significant (effect = 0.196, 95% CI [0.147, 0.246]).

### 3.4 Moderated mediation model

Based on the established mediation model, autonomy, control, and impersonal orientations were included as moderating variables. To test their effects on all paths of the mediation model, Model 59 of the PROCESS macro was used.

#### 3.4.1 Autonomy orientation

The scores of need support, intrinsic motivation, and autonomy orientation were centered. Autonomy orientation and needs support scores after centering were multiplied to obtain the interaction term scores. These scores were used to test intrinsic motivation and PERMA well-being. Next, the scores for intrinsic motivation and autonomy orientations, after centering, were multiplied to obtain interaction term scores. These scores were used to examine PERMA well-being. As shown in Table 6, the interaction term of autonomy orientation and needs support significantly affected intrinsic motivation in the first half of the path. In the direct path, the interaction term of autonomy orientation and needs support did not significantly predict PERMA well-being. In the second half of the path, the interaction term of autonomy orientation and intrinsic motivation did not significantly predict PERMA well-being. Therefore, the moderating effect of autonomy orientation was significant in the first half of the mediation model’s path but not in the other paths.

**Table 6:** Moderated mediation model of autonomy orientation.

Predictors	On IM			On PERMA		
	*β*	SE	*t*	*β*	SE	*t*
Constant	−0.007	0.012	−0.586	6.976	0.056	123.539
NsSu	0.169	0.014	12.404***	0.331	0.068	4.847***
IM				1.469	0.182	8.093***
AO	0.139	0.016	8.501***	0.212	0.075	2.804**
AO × NsSu	0.028	0.012	2.340*	0.057	0.062	0.912
AO × IM				0.279	0.182	1.538
R^2^	0.349			0.286		
F	110.978***			49.583***		

AO, autonomy orientation; NsSu, need support; IM, intrinsic motivation; PERMA, PERMA well-being.

* *p* < 0.05, ** *p* < 0.01, *** *p* < 0.001.

**Figure 4:**
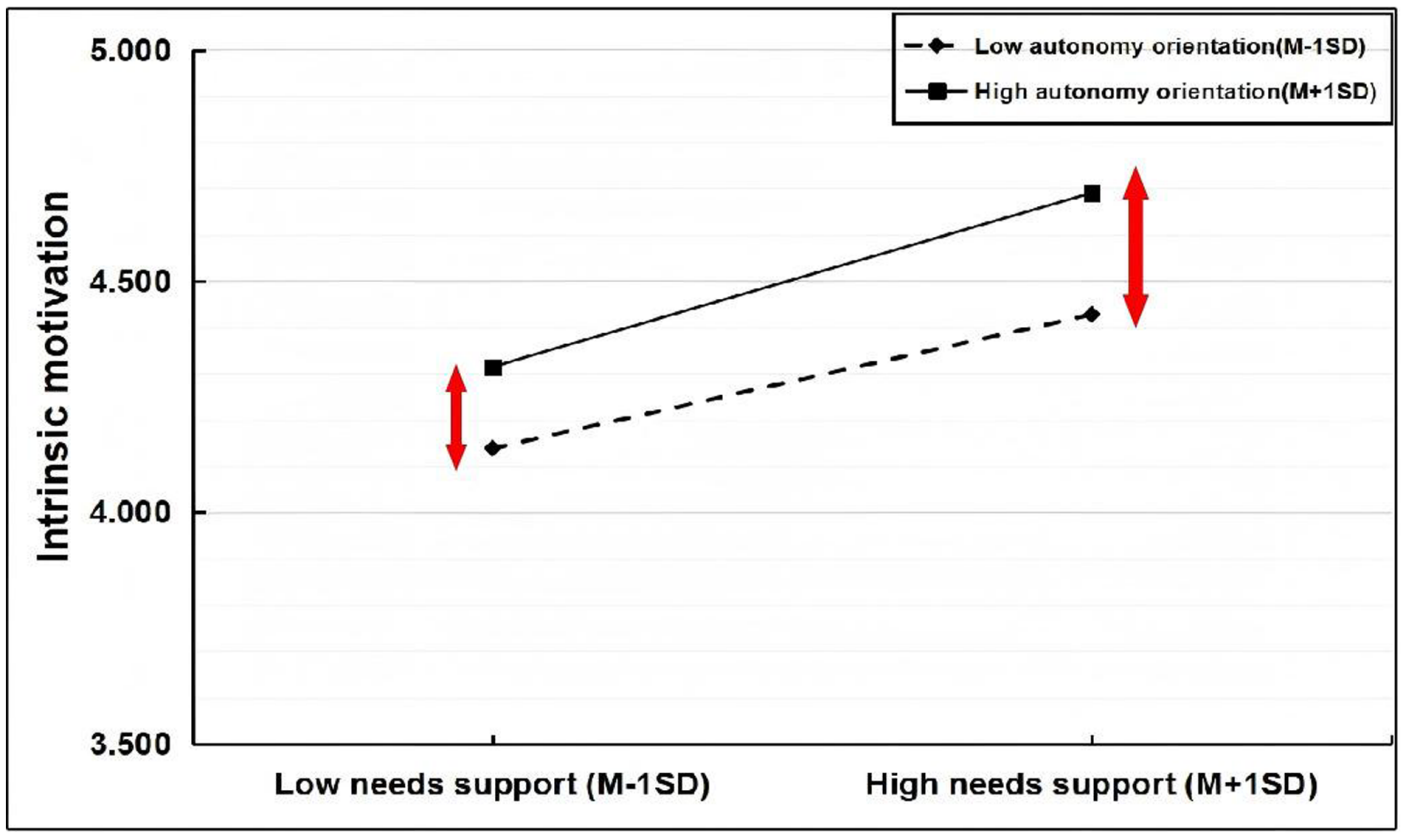
Plot of moderated effects of autonomy orientation.

Autonomy orientation was divided into high and low groups. The low group was defined as having values below M − 1SD, and the high group had values above M + 1SD. The moderating effect of autonomy orientation in the first half of the path was analyzed using a simple slope. The results are presented in Figure 4. With low autonomy orientation, the positive effect of needs support on intrinsic motivation was significant (simple slope = 0.147, *t* = 10.341, *p* < 0.001). With high autonomy orientation, the positive effect was stronger (simple slope = 0.192, *t* = 10.260, *p* < 0.001). In other words, as autonomy orientation increased, the positive predictive effect of needs support on intrinsic motivation also increased.

**Figure 5:**
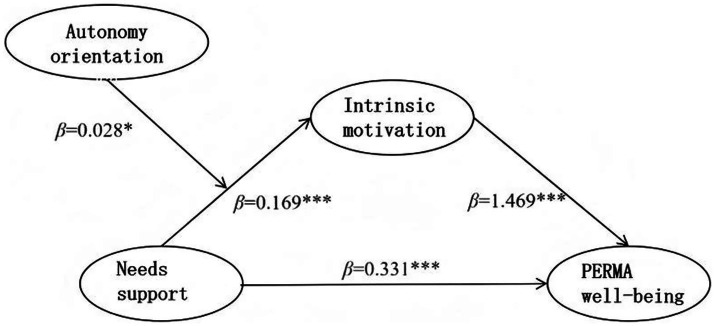
Path diagram of the validated moderated mediation of autonomy orientation.

As shown in Table 7 and Figure 5, the mediating effects were significant with both high and low autonomy orientations (95% CI did not contain 0). The mediating effect was stronger with high autonomy orientation (0.324, 95% CI [0.208, 0.463]) than with low autonomy orientation (0.184, 95% CI [0.101, 0.260]). Thus, as autonomy orientation increased, the mediation model effect also increased, supporting H2a.

**Table 7:** Moderating effect of autonomy orientation on the mediation model.

Mediator	Moderator levels (AO)		Effect	SE	95% CI	
					Lower limit	Upper limit
IM	M − SD	4.063	0.231	0.039	0.153	0.307
	M	4.853	0.266	0.038	0.195	0.344
	M + SD	5.643	0.301	0.053	0.206	0.414

AO, autonomy orientation; IM, intrinsic motivation.

#### 3.4.2 Control orientation

The predictor variables for needs support, intrinsic motivation, and control orientation were centered. The scores of control orientation and needs support after centering were multiplied to create the interaction term. Intrinsic motivation and PERMA well-being were examined. Moreover, intrinsic motivation and control orientation scores were multiplied to create the interaction term, and PERMA well-being was examined. As shown in Table 8, the interaction term of control orientation and needs support did not significantly affect intrinsic motivation in the first half of the path. In the direct path, the interaction term of control orientation and needs support did not significantly predict PERMA well-being. In the second half of the path, the interaction term of control orientation and intrinsic motivation did not significantly predict PERMA well-being. Therefore, the moderating effect of control orientation was not significant for all paths of the mediation model, rejecting H2b.

**Table 8:** Moderated mediation model for control orientation.

Predictors	On IM			On PERMA		
	*β*	SE	*t*	*β*	SE	*t*
Constant	−0.008	0.010	−0.765	6.992	0.069	101.935
NsSu	−0.019	0.013	−1.520	0.283	0.078	3.620***
IM				1.486	0.243	6.118***
CO	−0.239	0.009	−26.135***	−0.073	0.081	−0.903
CO × NsSu	−0.009	0.006	−1.443	0.055	0.045	1.209
CO × IM				−0.209	0.127	−1.649
*R* ^2^	0.654			0.274		
*F*	392.229***			46.895***		

CO, control orientation; NsSu, need support; IM, intrinsic motivation; PERMA, PERMA well-being.

* *p* < 0.05, ** *p* < 0.01, *** *p* < 0.001.

#### 3.4.3 Impersonal orientation

The values of the predictor variables for needs support, intrinsic motivation, and impersonal orientation were centered. The scores of the impersonal orientation and needs support after centering were multiplied to create the interaction term. Intrinsic motivation and PERMA well-being were tested separately. Subsequently, intrinsic motivation and impersonal orientation scores were centered and multiplied to create the interaction term, and PERMA well-being was examined. As shown in Table 9, the interaction term of impersonal orientation and needs support significantly affected intrinsic motivation in the first half of the path. In the direct path, the interaction term of impersonal orientation and needs support did not significantly predict PERMA well-being. In the second half of the path, the interaction term of impersonal orientation and intrinsic motivation did not significantly predict PERMA well-being. Therefore, the moderating effect of impersonal orientation was significant in the first half of the mediation model’s path but not in the other paths.

**Table 9:** Moderated mediation model for impersonal orientation.

Predictors	On IM			On PERMA		
	*β*	SE	*t*	*β*	SE	*t*
Constant	−0.020	0.010	−2.014	6.954	0.067	104.221
NsSu	−0.070	0.013	−5.222***	0.275	0.086	3.188**
IM				1.312	0.252	5.209***
IO	−0.300	0.011	−28.052***	−0.167	0.101	−1.645
IO × NsSu	−0.022	0.006	−3.754***	−0.024	0.048	−0.504***
IO × IM				−0.135	0.131	−1.027
*R* ^2^	0.679			0.276		
*F*	438.318***			47.294***		

IO, impersonal orientation; NsSu, needs support; IM, intrinsic motivation; PERMA, PERMA well-being.

* *p* < 0.05, ** *p* < 0.01, *** *p* < 0.001.

**Figure 6:**
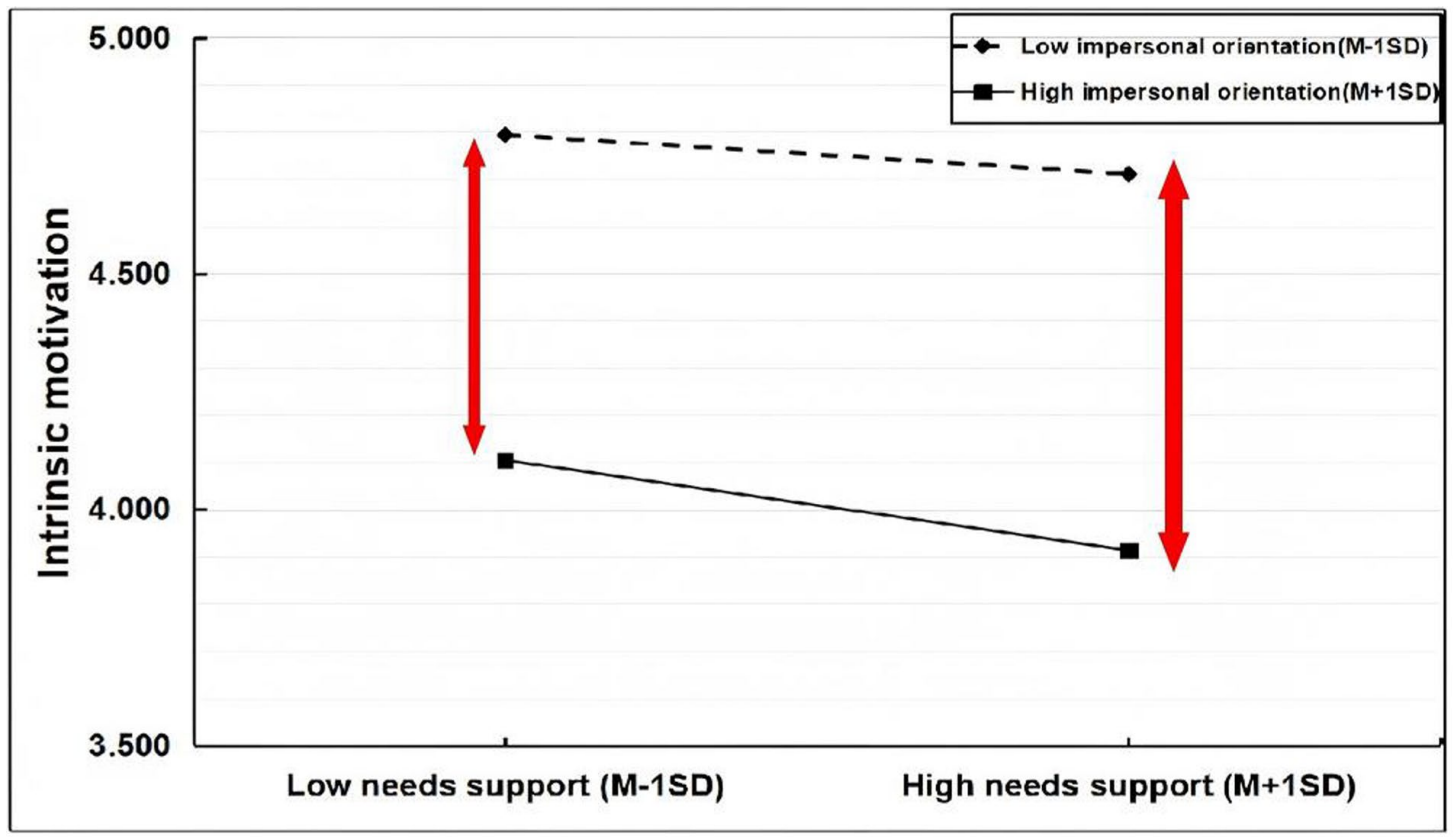
Plot of moderated effects of impersonal orientation.

Impersonal orientation values were grouped into high and low categories. Values below M − 1SD were designated as the low group, and values above M + 1SD were designated as the high group. A simple slope was used to analyze impersonal orientation in the first half of the path. The results are presented in Figure 6. After adding impersonal orientation, the predictive effect of needs support on intrinsic motivation became negative with low impersonal orientation (simple slope = −0.042, *t* = 2.588, *p* < 0.05) and more significant with high impersonal orientation (simple slope = −0.097, *t* = −6.916, *p* < 0.001). Thus, the positive predictive effect of needs support on intrinsic motivation weakened and became negative as impersonal orientation increased. It is noteworthy that the simple slope for needs support was negative at low levels of impersonal orientation. This pattern can occur in moderation analyses when the predictor and moderator are substantially correlated, as they were in this study (*r* = −0.75, see Table 2), and the interaction is significant. It indicates that the conditional effect of needs support on intrinsic motivation, when accounting for the strong main effect of impersonal orientation and the interaction, is not positive even at low levels of the moderator. This underscores the pervasive and overriding negative association between impersonal orientation and intrinsic motivation in this context.

**Figure 7:**
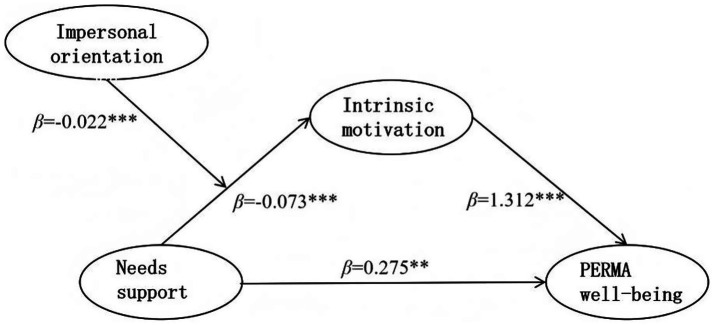
Path diagram of the validated moderated mediation of impersonal orientation.

Table 10 presents the mediating effect results moderated by impersonal orientation, and Figure 7 presents the path diagram. With both high and low impersonal orientation, the mediating effect changed from positive to negative. Thus, impersonal orientation significantly weakened the mediation model, transforming the mediating effect into a masking effect. Although the negative mediating effect was not significant with low impersonal orientation (−0.062, 95% CI [−0.140, 0.003], *p* > 0.05; 95% CI contained 0), it became significant with high impersonal orientation (−0.111, 95% CI [−0.281, −0.026]), supporting H2c.

**Table 10:** Moderating effect of impersonal orientation on the mediation model.

Mediator	Moderator levels (IO)		Effect	SE	95% CI	
					Lower limit	Upper limit
IM	M-SD	2.350	−0.066	0.036	−0.141	0.001
	M	3.589	−0.110	0.041	−0.198	−0.037
	M + SD	4.828	−0.153	0.061	−0.293	−0.055

AO, autonomy orientation; IM, intrinsic motivation.

## 4 Discussion

### 4.1 Interpretation of the mediating pathway and theoretical contribution

Consistent with SDT, our findings demonstrate that intrinsic motivation serves as a key mechanism in the relationship between PE teachers’ needs support and students’ multifaceted PERMA well-being. This extends the SDT literature by validating its core propositions within the integrated PERMA framework in a Chinese PE context (Ryan and Deci, 2020; Seligman, 2012). The partial mediation suggests that while fostering intrinsic motivation is crucial, needs support also enhances PERMA—potentially by directly fostering positive relationships with the teacher (relatedness), a sense of accomplishment (competence), and more volitional engagement (autonomy) (Germani and Palombi, 2022; Guo et al., 2023; Slemp et al., 2024). This underscores that a needs-supportive climate is a fundamental educational strategy that contributes to student flourishing in multiple ways.

### 4.2 Interpretation of the moderated mediation model

Our finding that autonomy orientation significantly moderated the first stage of the mediation model—specifically, by strengthening the positive relationship between needs support and intrinsic motivation—aligns with the SDT principle of motivational synergy (Deci and Ryan, 2000). Students with this orientation are predisposed to perceive needs support as congruent with their self, thereby more fully internalizing it and generating stronger intrinsic motivation. This amplified motivation likely facilitates a richer experience of the engagement, accomplishment, and meaning dimensions of PERMA, both in and outside of class. The strengthening effect of autonomy orientation delineates the scope of maximum returns for need-supportive teaching, highlighting that students predisposed to value self-direction benefit most profoundly from such an environment.

We examined the null effect of control orientation and its cultural context. The non-significant moderating role of control orientation appears to contradict some experimental studies (Hagger and Chatzisarantis, 2011) but can be contextualized. In the Chinese educational setting, where PE is mandatory and academic achievement is highly valued, control-oriented motivations (e.g., focusing on grades) may be normative. Consequently, in a generally supportive environment, needs support might still effectively satisfy psychological needs and promote well-being for these students, not necessarily by enhancing intrinsic motivation, but potentially by fostering more autonomous forms of extrinsic motivation (e.g., seeing personal value in the activity) (Zheng et al., 2023), which were not the focus of this study (Baard et al., 2004). Therefore, the non-significant finding for control orientation should be interpreted within this specific cultural and curricular context and may not generalize to educational settings where PE is elective or less grade-focused.

*The debilitating and reversing role of impersonal orientation*. The most critical finding concerns impersonal orientation, which significantly moderated the first stage, not only attenuating, but reversing the positive relationship between needs support and intrinsic motivation. This can be interpreted through the SDT lens of need frustration (Ryan and Deci, 2017). For these students, who feel incompetent and helpless, well-intentioned support may be perceived as pressure that highlights their inadequacy, thereby triggering a defensive response that further undermines their intrinsic motivation (Burgueño et al., 2023; González-Cutre et al., 2023). This aligns with findings that intrinsic motivation is strongly negatively correlated with amotivation and underscores a boundary condition for SDT: support can be counterproductive when it interacts with a predisposition for need frustration, as seen in other life domains (Malinowska and Tokarz, 2020). This effect critically defines the practical scope of SDT in PE, demonstrating that generalized need support is ineffective and even harmful for students high in impersonal orientation, necessitating a prior, tailored approach to address their core feelings of incompetence and amotivation.

It is noteworthy that the moderating effects were significant only for the first stage of the mediation model (needs support → intrinsic motivation). This may be because we used exercise-specific causality orientations as moderators, the influence of which is most directly relevant to the motivational processes within the PE context itself. In contrast, PERMA well-being is a broader, more distal outcome likely influenced by a wider array of factors beyond the specific exercise context.

This study tested a moderated mediation model grounded in the integrated frameworks of SDT and COT. In developing this model, we considered it against key theoretical alternatives. A direct-effect model was found to be less comprehensive, while a simple mediation model could not account for the critical individual differences captured by causality orientations. The final model, by contrast, most parsimoniously captures the complex interplay wherein the motivational pathway from need support to well-being is contingent upon students’ dispositional orientations.

### 4.3 Practical implications

The correlational evidence from this study provides a preliminary guide for need-supportive teaching in mandatory Chinese college PE: to tailor support to students’ causality orientations rather than apply a uniform approach (Hagger and Hamilton, 2020; Kruse et al., 2024). For students with high impersonal orientation, educators could focus on reducing helplessness by structuring tasks to ensure initial competence experiences and offering limited, structured choices to gradually foster a sense of autonomy (Behzadnia et al., 2022). For autonomy-oriented students, instructors might further support their self-directed tendencies by delegating leadership roles and incorporating self-paced learning opportunities. For control-oriented students, framing need-supportive strategies in terms of their valued outcomes (e.g., academic performance benefits) may help align external requirements with more internalized motivation. These suggestions are provisional and warrant validation through future intervention-based research.

## 5 Conclusion

This study demonstrated that the SDT-based motivational associations in PE is conditioned by students’ exercise causality orientations. We provide robust evidence that the commonly advocated needs-support approach can, counterintuitively, show a null or even negative association with intrinsic motivation among students with high impersonal orientations. This key finding underscores a critical boundary condition of SDT and shifts the pedagogical implication from merely providing support to strategically tailoring it based on students’ motivational dispositions. The integration of SDT and COT offers a more nuanced, person-centered framework for understanding and promoting well-being in mandatory educational settings like Chinese PE.

## 6 Limitations and future directions

Several limitations should be noted. First, the cross-sectional design precludes causal conclusions, warranting future longitudinal studies to verify temporal relationships. Second, the findings are based on Chinese university students in a mandatory PE system; their generalizability to other cultures, educational policies, or age groups requires further testing. Third, although a stratified sampling approach was used, recruitment through administrative channels may affect sample representativeness. Future research should employ more direct randomization.

Based on these limitations, future research could employ longitudinal designs such as cross-lagged panel models to establish causal relationships and trace how causality orientations interact with environmental support over time. Cross-cultural comparisons between educational systems with mandatory versus elective PE would help clarify how broader policies shape these motivational processes. Further investigation should examine additional mediators, such as basic psychological need satisfaction and frustration, particularly among students with control and impersonal orientations, to better explain the motivational sequence toward well-being.

## Data Availability

The original contributions presented in the study are included in the article/Supplementary material, further inquiries can be directed to the corresponding author.
