# Correction: Moderated mediation model of basic needs supports, intrinsic motivation, and PERMA well-being among college students in physical education: the moderating roles of exercise causality orientations

**DOI:** 10.3389/fpsyg.2026.1824966

**Published:** 2026-03-31

**Authors:** Liang Han, Man-man Li, Chong-yao Xiao

**Affiliations:** 1 Department of Social Sports, Henan Sport University, Zhengzhou, China; 2 School of Physical Education and Sport, Henan University, Kaifeng, China; 3 School of Physical Education, Zhengzhou Normal University, Zhengzhou, China; 4 School of Education and Health, Guilin Institute of Information Technology, Guilin, China

**Keywords:** causality orientation, intrinsic motivation, moderated mediation model, needs support, PERMA well-being, physical education

Figures 1–7 were in the wrong order.

**Figure 1:**
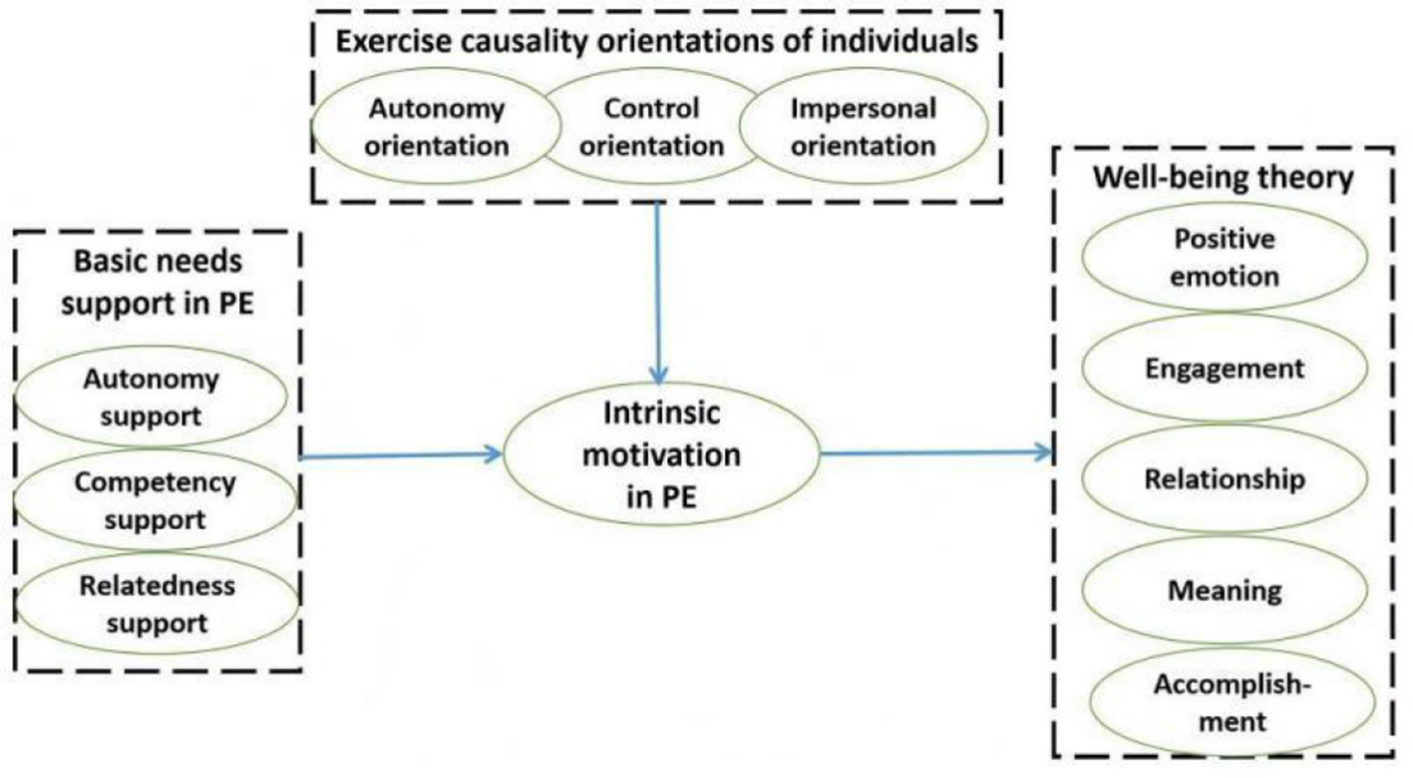
Theoretical model of this study.

**Figure 2:**
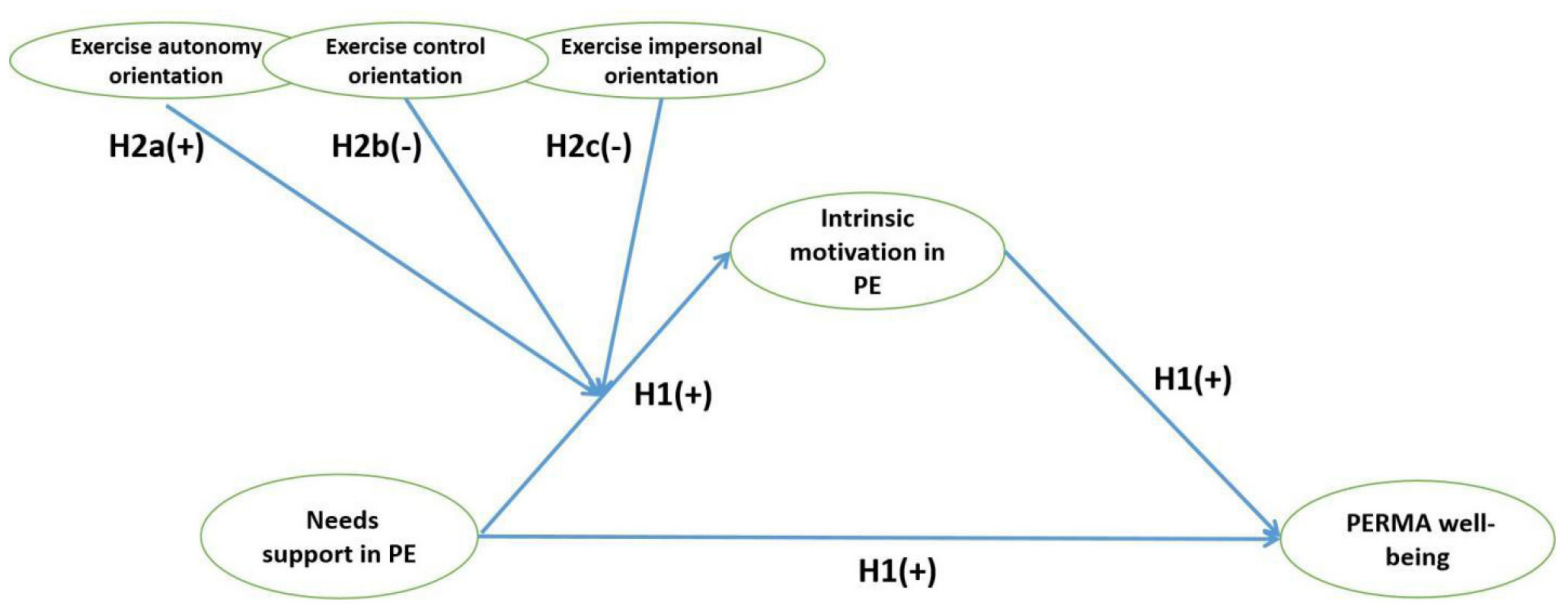
The overall hypothetical model.

**Figure 3:**
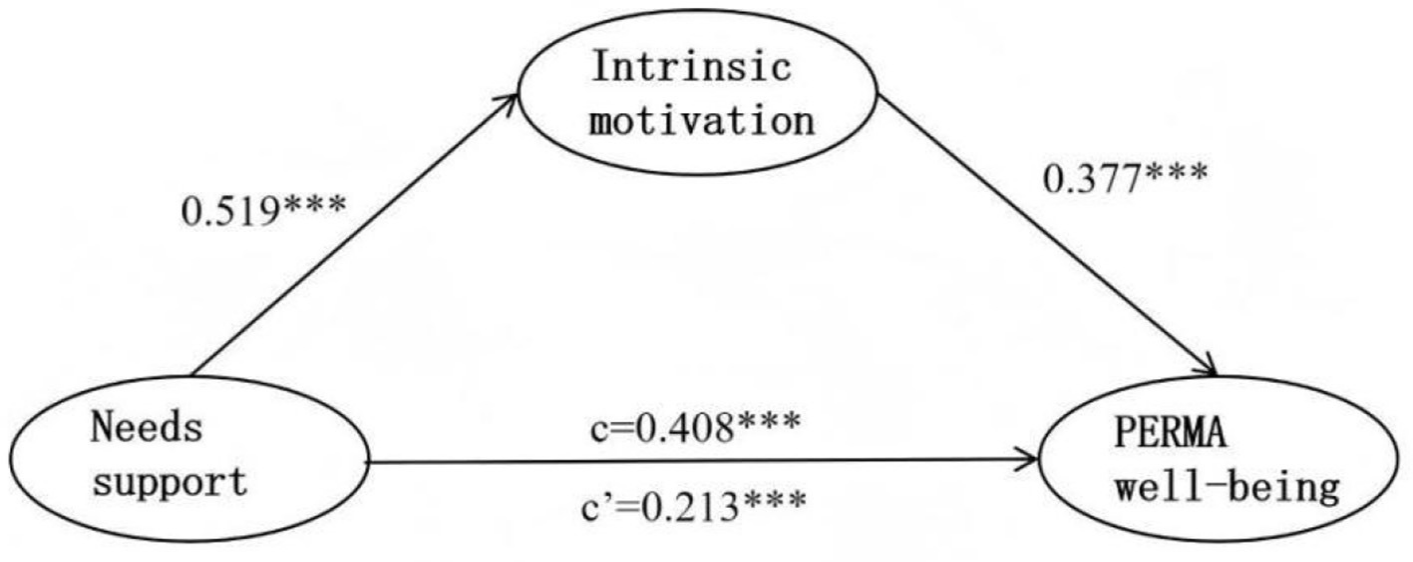
Diagram of the mediating effect test model.

**Figure 4:**
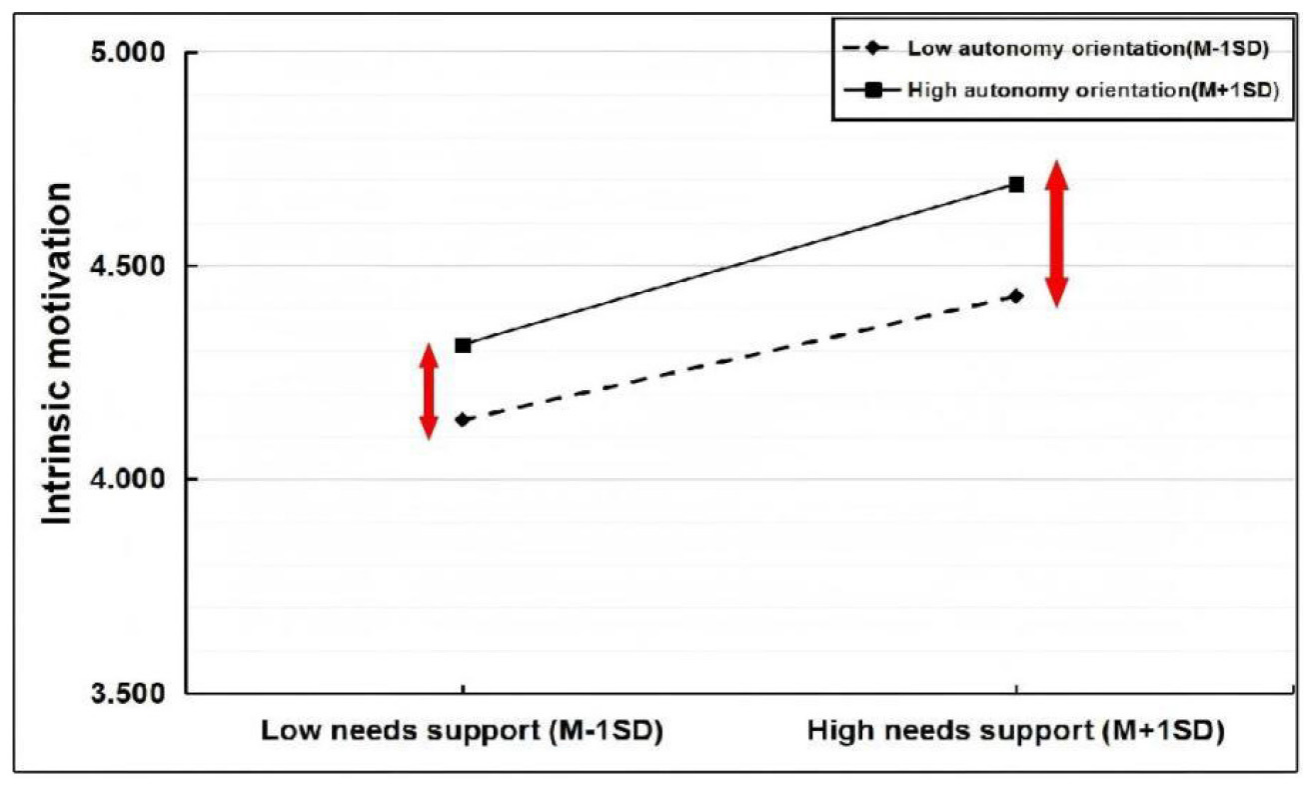
Plot of moderated effects of autonomy orientation.

**Figure 5:**
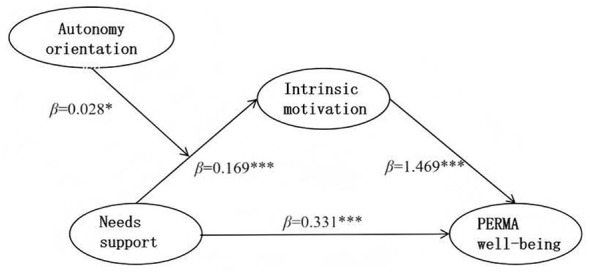
Path diagram of the validated moderated mediation of autonomy orientation.

**Figure 6:**
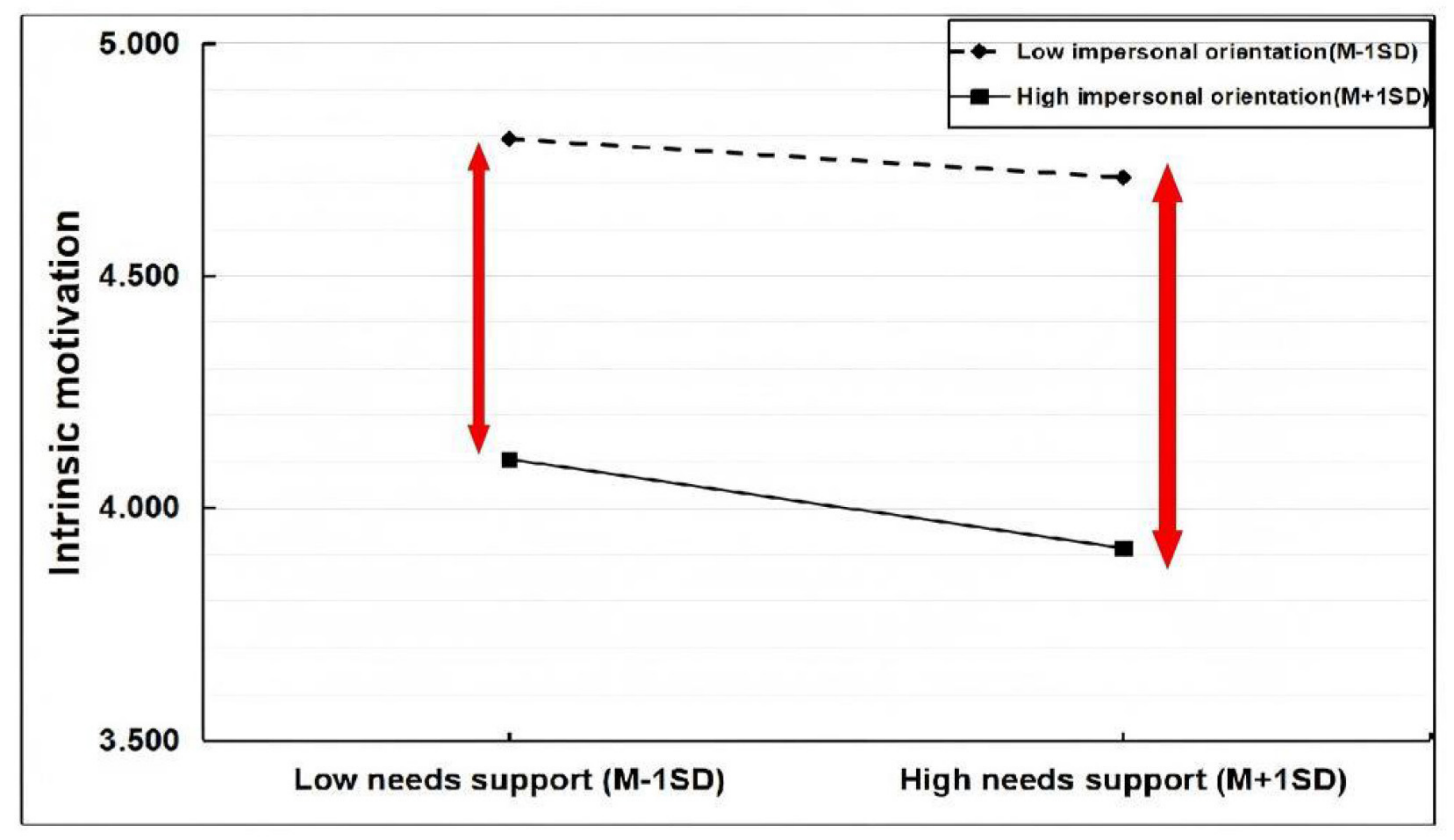
Plot of moderated effects of impersonal orientation.

**Figure 7:**
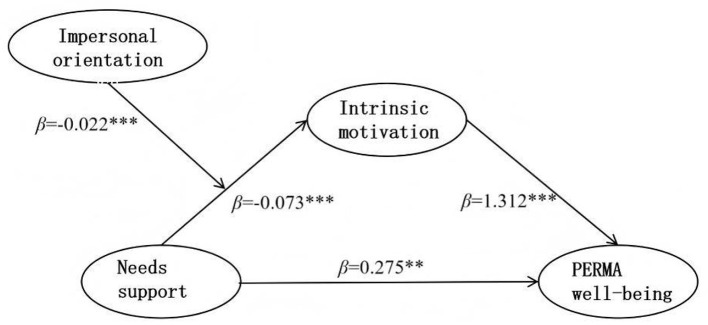
Path diagram of the validated moderated mediation of impersonal orientation.

The correct Figure 1 was erroneously given as Figure 4.

The correct Figure 2 was erroneously given as Figure 6.

The correct Figure 3 was erroneously given as Figure 5.

The correct Figure 4 was erroneously given as Figure 7.

The correct Figure 5 was erroneously given as Figure 1.

The correct Figure 6 was erroneously given as Figure 3.

The correct Figure 7 was erroneously given as Figure 2.

The original version of this article has been updated.

